# To compare the influence of blind insertion and up-down optimized glottic exposure manoeuvre on oropharyngeal leak pressure using SaCoVLM™ video laryngeal mask among patients undergoing general anesthesia

**DOI:** 10.1007/s10877-022-00930-1

**Published:** 2022-10-29

**Authors:** Chun-ling Yan, Yi-qi-yuan Zhang, Ying Chen, Zong-yang Qv, Ming-zhang Zuo

**Affiliations:** Department of Anesthesia, Institute of Geriatric Medicine, Beijing Hospital, National Center of Gerontology, Chinese Academy of Medical Sciences, Beijing, People’s Republic of China

**Keywords:** Oropharyngeal leak pressure, Blind insertion manoeuvre, Glottic exposure grading, SaCoVLM™ video laryngeal mask (VLM), Up-down optimized glottic exposure manoeuvre

## Abstract

To compare the potential influences of blind insertion and up-down optimized glottic exposure manoeuvre on the oropharyngeal leak pressure (OPLP) in using SaCoVLM™ video laryngeal mask (VLM) among patients undergoing general anesthesia. A randomized self-control study controlled was conducted to investigate the effect of two insertion techniques on OPLP. A total of 60 patients (male or female, 18–78 years, BMI 18.0–30.0 kg m^−2^ and ASA I–II) receiving selective surgery under general anesthesia were randomly recruited. After induction of anesthesia, the SaCoVLM™ was inserted by blind insertion manoeuvre. The glottic exposure grading(V1) of the SaCoVLM™ visual laryngeal mask and the OPLP(P1) were recorded. And the glottic exposure grading(V2) and OPLP(P2) of SaCoVLM™ were recorded again when the glottic exposure grading was optimal. The glottis exposure grading and OPLP were compared before and after different insertion manoeuver. The glottic exposure grading (V2) obtained by using up-down optimized glottic exposure manoeuvre was better than that obtained by using blind insertion manoeuvre (V1)(P < 0.001). The OPLP was significantly lower in the blind insertion manoeuvre (P1) than in the up-down optimized glottic exposure manoeuvre (P2) (32.4 ± 5.0 cmH_2_O vs. 36.3 ± 5.2 cmH_2_O, *P* < 0.001). In using SaCoVLM™, higher OPLP and better glottic exposure grading were achieved through up-down optimized glottic exposure manoeuvre, protecting the airway while real-time monitoring of conditions around the glottis, which significantly improves airway safety. Our results suggests that up-down optimized glottic exposure manoeuver may be a useful technique for SaCoVLM™ insertion.

*Trial registration*: ChiCTR, ChiCTR2000028802. Registered 4 January 2020, http://www.chictr.org.cn/ChiCTR2000028802.

## Background

**Fig. 1:**
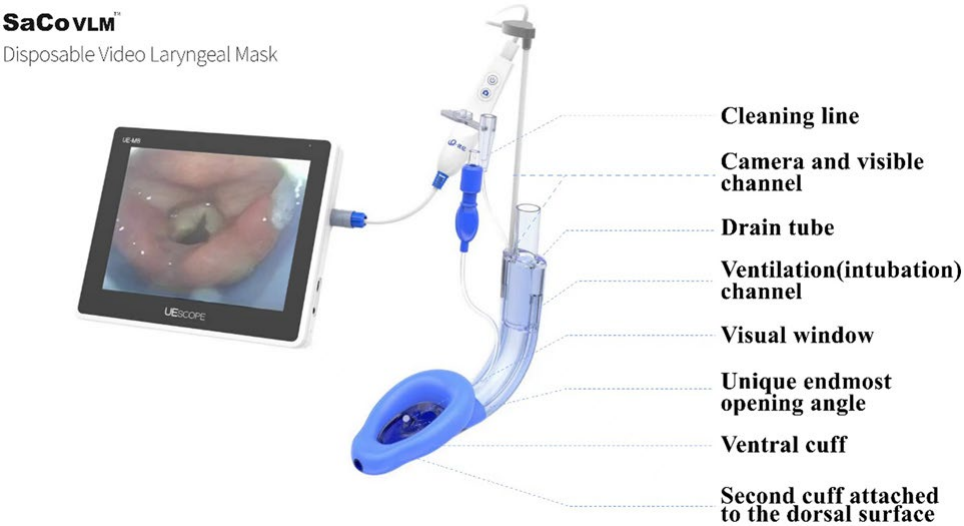
SaCoVLM™ disposable video laryngeal mask

SaCoVLM™ video laryngeal mask (SaCoVLM™ ZHEJIANG UE MEDICAL CORP. Add: No. 8, Youyi Road, Baita Economic Develop Zone, Xianju, Zhejiang, China) (Fig. 1) [1, 2] is a newly developed laryngeal mask, that combines visualization technology with the second generation LMA to rapidly and precisely expose the glottis and surrounding tissues. It is a device consisting of a visual channel, ventilation (intubation) channel, drain tube and other components with both data storage and rechargeable functions. A camera (electronic camera, focal length of the camera, 7 mm; field of view, 90° ± 13.5%) on the right side of the ventral cuff can be connected with the screen, and inserted into the visual channel for adjusting the insertion of SaCoVLM™ in a real-time manner.

The LMA CTrach™ [3] was the first visual laryngeal mask introduced for clinical use, followed by Totaltrack™ [4]. Both of the VLMs are designed for intubation and were used to guide tracheal intubation [5–8]. As the ventilator tube of the two types of VLMs were relatively rigid and were single-tube, they were not suitable for maintaining ventilation. Therefore, there was no literature describing the use of these two types of VLMs to maintain ventilation and seal pressure. SaCoVLM™ is a dual-tube laryngeal mask for maintenance ventilation with a visual function. OPLP could be used to identify the success of positive pressure ventilation, but also measure the airway protection [9, 10]. This study aims to explore the influence of blind insertion and up-down optimized glottic exposure manoeuvre on the OPLP in using SaCoVLM™ among patients undergoing general anesthesia. Our findings will provide reference for identifying the optimal insertion method of SaCoVLM™.

## Clinical data and methods

### Subjects

This is a randomized self-controlled study approved by the Ethics Committee of Clinical Research of Beijing Hospital (No. 2019BJYYEC-236-02) and registered in the China Clinical Trial Registration Center (No. ChiCTR2000028802 Date 2020.01.04). This study adheres to the Consolidated Standards of Reporting Trials (CONSORT) guidelines. The study was approved by the appropriate Institutional Review Board (IRB), and written informed consent was obtained from all subjects. From February 2020 to December 2020, a total of 60 patients receiving general, obstetrics and gynecological and urological surgeries were randomly recruited. For all patients, general anesthesia was performed in the supine position with the SaCoVLM™. Inclusion criteria: male or female; 18–78 years; BMI 18.0–30.0 kg/m^2^; ASA I–II. Exclusion criteria: Severe respiratory diseases; mouth opening < 2 cm; high risks of reflux of gastric contents and aspiration (including non-fasting, morbidly obese, gestational week > 14 weeks, frequent gastroesophageal reflux, intestinal obstruction and hiatal hernia); contraindications to the use of laryngeal mask airways; Laryngeal mask insertion failed.

### Anesthesia methods

Patients were routinely fasted for 8 h and abstained from drinking for 6 h. After entering the operating room, an intravenous catheter was inserted into a peripheral vein, and their electrocardiogram (ECG), heart rate (HR), blood pressure (BP), oxygen saturation (SpO_2_), end-expiratory carbon dioxide (P_ET_CO_2_), and bispectral index (BIS) were monitored by Drager monitors (Draeger Medical Inc, 3135 Quarry Road Telford, PA, USA). The size of SaCoVLM™ was selected based on the body weight of the patient: Size 3 for adults 30–50 kg; Size 4 for adults 50–70 kg and Size 5 for adults 70–90 kg. Before placement, the cuff was emptied and flattened. The back of the laryngeal mask was lubricated with lidocaine hydrochloride gel. 5 L/min of 100% oxygen was preoxygenated for 5 min before anesthesia induction. Intravenous induction was performed (sufentanil 0.2–0.5 ug/kg, propofol 2 mg/kg, and cis-atracurium 0.2 mg/kg), and mask ventilation was performed till BIS was below 60. After mask ventilation was smooth and mandibular joints were relaxed, the SaCoVLM™ was inserted. And then the SaCoVLM™ was inserted using the blind insertion manoeuvre as previously described by Brain et al. [11]. The glottis exposure grading (V1) was recorded and the airway seal pressure (P1) was measured. The anesthesia machine was connected for mechanical ventilation in the intermittent positive pressure ventilation (IPPV) mode with the following parameters: oxygen flow, 2.5 L min^−1^; tidal volume, 6 ml kg^−1^; inspiration/expiration (I/E) ratio, 1:2; ventilation frequency, 12–15 times/min; positive end-expiratory pressure (PEEP), 5 cmH_2_O; P_ET_CO_2_, 35–45 mmHg. After 5 min of maintenance of ventilation, the glottic exposure grading (V2) and OPLP (P2) were recorded again using up-down optimized glottic exposure manoeuver.

Blind insertion manoeuvre: briefly, the anesthesiologist held the distal end of the ventilation tube and let the SaCoVLM™ slide down the palatopharyngeal curve along midline in the mouth, until the front end of the SaCoVLM™ was inserted into the hypopharyngeal cavity, feeling an obvious resistance. Then, the cuff was inflated to 60 cmH_2_O (1 cmH_2_O = 0.098 kpa) detected by a hand-held manometer (VBM, SULZ, GERMANY).

Up-down optimized glottic exposure manoeuver: in brief, the anesthesiologist placed the VLM in a semi-inflated state(Internal pressure of capsule < 60 cmH_2_O), holding the distal end of the ventilation tube and pulling the SaCoVLM™ out 2 to 3 cm along the palatopharyngeal curve, and then re-inserting it into the hypopharyngeal cavity along the palatopharyngeal curve. In this process, the best position of glottic exposure grading was found. At the best position of glottis exposure grading, the SaCoVLM™ was inflated with a pressure of 60 cmH_2_O (1 cmH_2_O = 0.098 kpa) detected by a hand-held manometer (VBM, SULZ, GERMANY) and fixed.

### Measurement of OPLP

Criteria of successful SaCoVLM™ insertion: normal tidal volume ventilation with chest undulation and no air leakage sound, and more than two continuous P_ET_CO_2_ waveforms observed by capnography suggested satisfatory. A positive result of the suprasternal pit gel test confirmed that the gastroesophageal drainage tube of SaCoVLM™ was correctly aligned with the esophagus. Subsequently, OPLP was measured using the manometric stability method [10, 12]. Briefly, the intracuff pressure of SaCoVLM™ was adjusted to 60 cmH_2_O, and the fresh gas flow rate was adjusted to 3 L min^−1^, the pressure valve was adjusted to 40 cmH_2_0, and mechanical ventilation was switched to manual ventilation. When the sound of air leakage was heard, the pressure was gauged as the OPLP. For the sake of safety, the maximum OPLP was set to be 40 cmH_2_O [13]. The OPLP by blind insertion (P1) was recorded when a sound of leak was heard over the mouth. This view of the glottis was obtained as V1. To obtain a clear view of the glottis, an up-down manoeuvre was performed by slowly withdrawing the SaCoVLM™ and re-inserting it alongside the palatopharyngeal curve. Once the best exposure field was obtained (V2), the SaCoVLM™ was fixed and the intracuff pressure was adjusted to 60 cmH_2_O. The OPLP was measured by the manometric stability test as P2.

### Glottic exposure grading

**Fig. 2:**
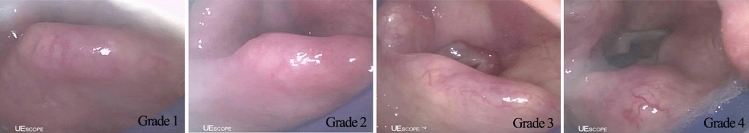
SaCoVLM™ Glottic exposure grades. Grade 1: visualization of the lateral part of the right aryepiglottic fold and part of the laryngeal inlet, and the ventilation was good; Grade 2: visualization of the bilateral aryepiglottic fold and part of laryngeal inlet, and the ventilation was good; Grade 3: visualization of all laryngeal inlet and posterior glottis; Grade 4: visualization of the whole glottis

In this study, the glottic exposure by the SaCoVLM™ was classified into 4 grades as follows (fig. 2) [2]: Grade 1, visualization of the lateral part of the right aryepiglottic fold and part of the laryngeal inlet, and the ventilation was good; Grade 2, visualization of the bilateral aryepiglottic fold and part of laryngeal inlet, and the ventilation was good; Grade 3, visualization of all laryngeal inlet and partial glottis; Grade 4, visualization of the whole glottis. In the same patient, the position of the SaCoVLM™ was adjusted by the up-down maneuver, and the glottis exposure field obtained at the highest grade of 1–4 was regarded as the best exposure field for the patient. All procedures were performed by the same anesthetist with at least 5-year clinical experiences.

### Maintain anesthesia

Anesthesia was maintained by the target controlled infusion (TCI) of 2.5–3.5 µg ml^−1^ propofol and 3–4 ng ml^−1^ remifentanil with BIS ranging from 40 to 60. During the maintenance of anesthesia, cisatracurium was intermittently administrated. TCI of propofol and remifentanil was discontinued at the time of skin closure, and then antagonists of muscle relaxants were applied. The SaCoVLM™ was removed when spontaneous recovery of consciousness was obtained. The patient was transferred to the Post Anesthesia Care Unit (PACU).

### Data collection

The primary variables: OPLP (P1) obtained by traditional blind insertion manoeuvre and the OPLP (P2) obtained by up-down optimized glottic exposure manoeuvre. The secondary variables: the glottic exposure grading (V1) with traditional blind insertion manoeuvre and the glottic exposure grading (V2) with up-down optimized glottic exposure manoeuvre.

### Statistical analysis

According to the data of 10 pre-experimental cases, the *t* test of two samples showed that the sample size was 60. All data were analyzed using SPSS 26.0 statistical software for data analysis, and measurement data were expressed as Mean ± standard deviation (x ± s). X^2^ test was used in the graded data. The OPLP obtained by two different manoeuvre was tested by paired sample *T* test; P < 0.05 was considered a significant difference.

## Results

A total of 60 eligible patients were recruited in this study, involving 14 (23.3%) males and 46 (76.7%) females. Their average age, height, body weight and body mass index (BMI) were 51.2 years, 162.7 cm, 63.1 kg and 23.0 kg m^−2^, respectively. Baseline characteristics of them were listed in Table 1.

The OPLP (P2) obtained by the up-down optimized glottic exposure manoeuvre was significantly higher than that by blind insertion manoeuvre (P1) (*P* < 0.001, Table 2).

Compared with blind insertion manoeuvre, the up-down optimized glottic exposure manoeuvre obtained a significantly better view of the glottis (*P* < 0.001, Table 3).

## Discussion

In this study, we compared the effect of different insertion techniques on the oropharyngeal seal pressure of the laryngeal mask when using SaCoVLM™. The results showed that the oropharynx leak pressure was higher when the optimal glottic exposure was obtained by using up-down optimized glottic exposure manoeuvre. Although supraglottic airway devices (SADs) have many desirable features, they are nevertheless inserted in a similar ‘blind’ way as their 1st generation predecessors. Clinicians mostly still rely entirely on subjective indirect assessments to estimate correct placement which supposedly ensures a tight seal. Malpositioning and potential airway compromise occurs in more than half of placements. Vision-guided insertion can improve placement [14]. In patients operated with CTrachTM or Totaltrach VLM, the operator will look for the glottis during placement and adjust the VLM to best expose the field of glottis. However, these two VLM are designed for intubation purposes, and the ventilation tube is thick and hard, not suitable for prolonged maintenance ventilation [8]. Therefore, there are no relevant studies on the OPLP of VLM under different glottic exposure fields.

SaCoVLM™ combines the features of the second-generation SAD, long-term ventilation and visualization of the glottis. It has been shown that SaCoVLM™ can be used to maintain ventilation in patients under general anesthesia [2]. Whether the SAD can obtain good counterpoint and OPLP is the key to the clinical airway management of the SAD. OPLP can not only identify the success of positive pressure ventilation, but also measure the degree of airway protection [15]. Kumar CM et al. pointed out that the accuracy of LMA placement can be determined by clinical signs such as oropharyngeal sealing pressure [12]. High oropharyngeal sealing pressure are desirable as they indicate the feasibility of positive pressure ventilation and the likelihood of successful supraglottic airway placement [10, 13, 15–19]. The SaCoVLM™ can observe glottic exposure due to its visual function, and can optimize glottic exposure by using the up-down manoeuvre. This study shows that when using the SaCoVLM™, a higher OPLP can be achieved (P2 > P1) when the glottis is exposed in the optimal position compared with the traditional blind insertion manoeuvre (V2 > V1). Traditional blind insertion manoeuvre may lead to SAD being placed too deep and the glottis exposure field is not optimal, thus affecting the sealing effect of SAD on the laryngeal inlet, so the OPLP is relatively low. In addtion, a case report described arytenoid dislocation after SAD insertion by the blind insertion manoeuvre, because of the too deep insertion of the SAD [20]. Optimizing glottic exposure placement by up-down manoeuvre can avoid placing the SAD too deep.

In the present study, 13 (21%) patients demonstrated the same grade of glottic view either by the blind insertion manoeuvre or the up-down optimized glottic exposure manoeuvre, involving 4 cases with grade 2, 4 with grade 3 and 5 with grade 4. Therefore, we believed when using the traditional blind insertion technique of SAD can not immediately obtain an optimal exposure of the glottis, and a satisfactory OPLP. To avoid airway damage, an upper limit of 40 cmH_2_O was set when measuring the OPLP. In the traditional blind insertion manoeuvre, 14 cases were stopped when the OPLP reached 40 cmH_2_O, while in the optimized glottic exposure manoeuvre, 30 cases were stopped when the OPLP reached 40 cmH_2_O. Therefore, in theory, the OPLP of P2 is higher than that of P1 by more than 4 cmH_2_O, so it is necessary to adjust the glottis exposure to obtain the best OPLP in the visible state.

The camera of the SaCoVLM™ is located on the right side of the opening of the ventilation channel in the ventral cuff, which differs from the upper location of the LMA CTrach™ [8]and the Totalrach VLM [21]. Therefore, our conclusion is only limited in the use of the SaCoVLM™.

The Difficult Airway Society (DAS) guidelines [22] on the management of unanticipated difficult intubation in adults considers ‘blind’ airway management techniques unreliable and associated with a high incidence of airway trauma. Studies have pointed out that all SADs advocates visual verification to eliminate misalignment [23].VLM, such as SaCoVLM™, can reduce complications by allowing visual confirmation of the location of SAD. At the same time obtain higher OPLP.

## Conclusion

Up-down optimized glottic exposure manoeuvre should be used in SaCoVLM™, which can obtain higher OPLP which related to better airway protection, preventing air leaks. At the same time, a good glottic exposure field can provide a good condition for direct-vision tracheal intubation and real-time monitoring of periglottic conditions during general anesthesia. Our results suggests that it may be a useful technique for SaCoVLM™ insertion.

**Table 1:** Demographic data

Indexes	$\overline{{\text{v}}}$ ± s/%
Sex (n)	
Male	14 (23.3%)
Female	46 (76.7%)
Age (years)	51.2 ± 11.4
Height (cm)	162.7 ± 5.5
Body weight (kg)	63.1 ± 8.0
BMI (kg·m^−2^)	23.0 ± 1.4
ASA	
I	34 (56.7%)
II	26 (43.3%)
Surgery type	
General surgeries	11 (18.3%)
Obstetrics and gynecology surgeries	45 (75.0%)
Urology surgeries	4 (6.7%)
Thyromental distance(mm)	8.7 ± 1.0
Mouth opening (mm)	4.2 ± 0.5
Upper lip bite test	
1	30(50%)
2	30(50%)
3	0(0%)

*ASA* American society of anesthesiologists physical status, *BMI* Body Mass Index

**Table 2:** OPLP values obtained through two placement manoeuvre in using the SaCoVLM™

	OPLP (cmH_2_O)	*P*
P1	32.4 ± 5.0	0.000
P2	36.3 ± 5.2	

*P1* the OPLP obtained by the blind insertion manoeuvre, *P2* the OPLP obtained by the up-down optimized glottic exposure manoeuvre

**Table 3:** Grades of the glottis views through two placement manoeuvre in using the SaCoVLM™

	Grade 1	Grade 2	Grade 3	Grade 4	*P*
V1 (n, %)	28 (46.7%)	15 (25.0%)	10 (16.7%)	7 (11.7%)	0.000
V2 (n, %)	0 (0%)	6 (10.0%)	18(30.0%)	36 (60%)	

## Data Availability

The datasets used and/or analyzed during the current study are available from the corresponding author on reasonable request.

## Abbreviations

SaCoVLM™: SaCoVLM™ video laryngeal mask
ECG: Electrocardiogram
HR: Heart rate
BP: Blood pressure
Sp0_2_: Oxygen saturation
BIS: Bispectral index
P_ET_CO_2_: End-tidal carbon dioxide
ASA: American Society of Anesthesiologists
BMI: Body mass index
MAP: Mean arterial pressure
LMA: Laryngeal mask airway
IPPV: Intermittent positive pressure ventilation
I/E: Inspiration/expiration ratio
PEEP: Positive end-expiratory pressure
TCI: Target controlled infusion
PACU: Post Anesthesia Care Unit
